# Case Report: Idiopathic hypereosinophilic syndrome presenting with gastrointestinal involvement mimicking IBD: a diagnostic challenge

**DOI:** 10.3389/fmed.2025.1595193

**Published:** 2025-08-01

**Authors:** Johnny Amer, Fathalla Noori, Dareen Hamdan, Turkey Mokhaimar, Ahmad Salhab

**Affiliations:** ^1^Department of Allied and Applied Medical Sciences, Faculty of Medicine and Allied Medical Sciences, An-Najah National University, Nablus, Palestine; ^2^Department of Biomedical Sciences and Basic Clinical Skills, Faculty of Medicine and Allied Medical Sciences, An-Najah National University, Nablus, Palestine

**Keywords:** hypereosinophilic syndrome, eosinophilia, idiopathic HES, asthma, eosinophilic infiltration, gastrointestinal symptoms

## Abstract

**Background:**

Idiopathic hypereosinophilic syndrome (iHES) is a rare hematologic condition characterized by persistent, unexplained eosinophilia and organ involvement. Its diagnosis is challenging due to overlapping features with other eosinophilic and inflammatory gastrointestinal disorders.

**Case presentation:**

We report a case of a 44-year-old male with a history of asthma who presented with chronic epigastric pain, rectal bleeding, and significant weight loss. Initial investigations, including elevated CRP and fecal calprotectin, suggested inflammatory bowel disease, and treatment was initiated accordingly. However, symptoms persisted, and further evaluations revealed marked eosinophilic infiltration in gastric and colonic biopsies, raising suspicion for eosinophilic gastroenteritis. Repeat endoscopy showed giant gastric folds with significant eosinophilic infiltration (>120 eosinophils/HPF). Imaging demonstrated gastrointestinal wall thickening, biliary involvement, and incidental pulmonary nodules. Bone marrow biopsy revealed preserved trilineage hematopoiesis with prominent eosinophilia. Infectious, autoimmune, allergic, and neoplastic causes were systematically excluded. Cytogenetic testing was negative for PDGFRA, PDGFRB, and FGFR1 mutations, ruling out clonal eosinophilic disorders. Based on persistent peripheral eosinophilia, histologic evidence of tissue infiltration, and exclusion of secondary or clonal causes, a diagnosis of iHES was established in accordance with WHO 2024 criteria. The patient started on systemic corticosteroids, achieving partial symptom relief. Due to relapse during steroid tapering, azathioprine was added as a steroid-sparing agent. Ongoing monitoring was planned with consideration of biologic therapy for future relapses.

**Conclusion:**

This case illustrates the diagnostic complexity of iHES presenting with gastrointestinal involvement mimicking inflammatory bowel disease. It highlights the importance of a structured diagnostic approach, including repeated tissue evaluation and hematologic assessment, in differentiating iHES from other eosinophilic and inflammatory disorders.

## 1 Introduction

Hypereosinophilic syndrome (HES) is a rare disorder characterized by persistent eosinophilia (>1,500 cells/μL) and eosinophil-mediated organ damage, often affecting the heart, lungs, skin, and nervous system (1–3). It is broadly categorized into primary (clonal), secondary (reactive), and idiopathic (IHES) forms (4). Primary HES typically results from myeloproliferative mutations, while secondary HES is associated with conditions such as parasitic infections, autoimmune diseases, or malignancies (5–7). IHES is diagnosed when no underlying cause is identified despite extensive evaluation (8). The disease’s presentation can mimic other eosinophilic conditions, contributing to diagnostic delays and misclassification (9). Symptoms are diverse and may include fatigue, rash, respiratory distress, neuropathy, or cardiac dysfunction (10, 11). Early diagnosis and prompt intervention are crucial in preventing irreversible organ damage (12). First-line treatment involves systemic corticosteroids, while immunosuppressants, tyrosine kinase inhibitors, or biologics may be required for refractory or specific HES subtypes (13–15).

## 2 Case presentation

A 44-year-old male with a history of asthma managed on Montelukast and Theophylline presented with a 4-month history of chronic, worsening epigastric pain. The pain was continuous, dull, and partially relieved by esomeprazole. Initially, there were no other gastrointestinal or systemic symptoms, and the pain was managed conservatively under the impression of gastritis, providing only temporary relief.

Over time, his symptoms evolved, and he developed rectal bleeding, alternating constipation and diarrhea, and unintentional weight loss of approximately 8 kg (about 10% of his body weight) over three months. Given this constellation of symptoms, further evaluation was undertaken. On physical examination and initial blood work, his white blood cell count was within normal limits; specifically, the total white blood cell (WBC) count was 8.2 × 10^9^/L. Eosinophils accounted for 19% of the total cell population in the differential count. To estimate the absolute eosinophils count, we applied a proportional calculation using the WBC count as a reference: Eosinophils count = WBC count × eosinophils percentage = 8.2 × 10^9^/L × 0.19 = 1.56 × 10^9^/L.

An abdominal ultrasound revealed diffuse colonic wall thickening and mesenteric lymphadenopathy, especially in the right iliac fossa. The patient was referred to gastroenterology. Due to elevated fecal calprotectin and C-reactive protein (CRP), an initial working diagnosis of inflammatory bowel disease (IBD) was considered. Endoscopic evaluation showed patchy erythema in the stomach and duodenum, and he started on corticosteroids, azathioprine, and mesalazine. However, biopsy findings at that time indicated only peptic ulceration with reactive inflammation, which did not fulfill the histological criteria for Crohn’s disease.

Approximately three months after the initial endoscopic evaluation, due to the progression of symptoms, a repeat endoscopy was performed in July 2024, which revealed giant gastric folds, particularly in the antrum and peri-pyloric areas, raising suspicion for a potential infiltrative pathology. Colonoscopy demonstrated colonic hyperemia and congestion without ulceration or mass lesions. Histopathological examination of both gastric and colonic biopsies revealed marked eosinophilic infiltration, exceeding 120 eosinophils per high-power field (HPF) in the lamina propria and submucosa, particularly prominent in the antral and ascending colonic mucosa. Mast cell aggregates were also observed in association with the eosinophilic infiltrates, raising suspicion for eosinophilic gastroenteritis.

**Figure 1:**
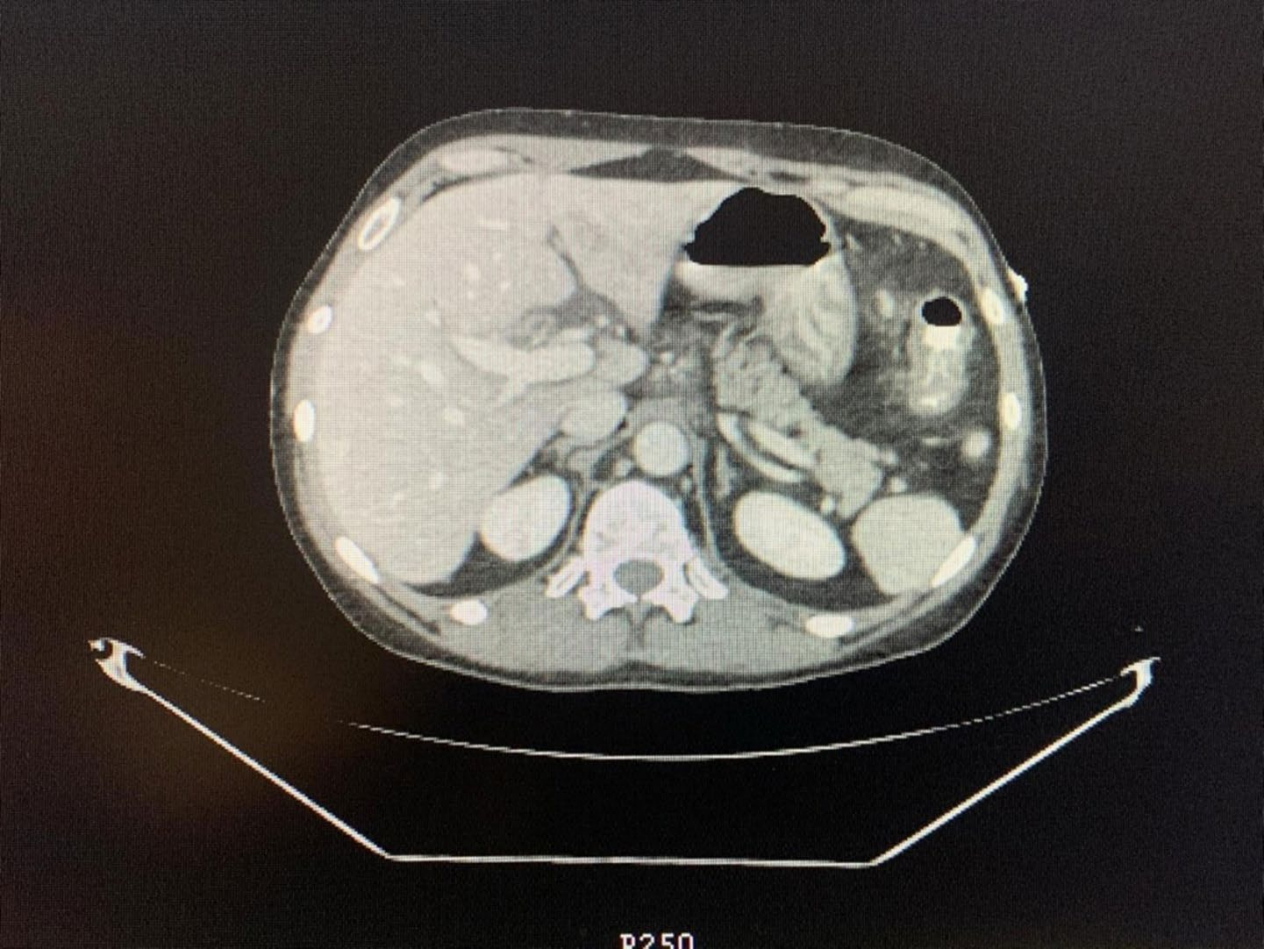
Abdominal CT scan: axial computed tomography (CT) image of the abdomen showing key anatomical structures.

To further explore systemic involvement and exclude other causes, abdominal CT imaging revealed diffuse thickening of the gastric and proximal duodenal walls, common bile duct involvement, bilateral lung nodules, and bronchiectasis (Figure 1). These findings, along with worsening abdominal symptoms, vomiting, and persistent weight loss, prompted more comprehensive investigation. These findings support a non-clonal eosinophilic process, consistent with idiopathic hypereosinophilic syndrome (iHES) following exclusion of secondary and neoplastic causes.

Repeated complete blood counts demonstrated persistent eosinophilia, with absolute eosinophil counts of 1800 cells/μL on multiple occasions over a 6-week period, despite normal total white blood cell and platelet counts. These values exceeded the 1,500 cells/μL threshold required for diagnosing hypereosinophilic syndrome.

Given ongoing peripheral and gastrointestinal eosinophilia and unclear etiology, a hematology consult was initiated. A bone marrow biopsy and aspiration were performed. (1) Bone marrow aspirate: The estimated myeloid-to-erythroid (M: E) ratio was 3:1. The erythroid precursors are quantitatively normal. The erythroid maturation is normoblastic. The granulocytic precursors are quantitatively normal with normal maturation, but with increased eosinophilic precursors. Blasts are not increased. Megakaryocytes, lymphocytes, and plasma cells were quantitatively normal. (2) Bone marrow biopsy: The core biopsy (2.7 cm) revealed slightly hypocellular marrow (cellularity 45%) with preserved trilineage hematopoiesis. Erythroid and granulocytic precursors appeared normal, and granulocytic maturation was intact, but with prominent eosinophilic precursors. Megakaryocytes, lymphocytes, and plasma cells were quantitatively normal. Iron staining showed adequate stainable iron, and no ringed sideroblasts were seen. These findings ruled out chronic eosinophilic leukemia and other myeloproliferative neoplasms, supporting the diagnosis of idiopathic HES. In addition, based on these findings and after ruling out Crohn’s disease, eosinophilic gastroenteritis (secondary causes), vasculitis, infections, and neoplasia, the diagnosis of iHES was made. This diagnosis is characterized by persistent blood and tissue eosinophilia without an identifiable secondary cause, with potential multi-organ involvement.

### 2.1 Comprehensive diagnostic evaluation for hypereosinophilia

iHES is a diagnosis of exclusion, necessitating a structured and detailed workup to rule out secondary (reactive) and clonal (primary) causes of eosinophilia (1–4). Following the 2024 World Health Organization (WHO) classification and international consensus guidelines for eosinophilic disorders (16), the following evaluations were systematically performed for this patient.

#### 2.1.1 Infectious causes

Infectious etiologies were excluded through comprehensive stool analysis, which was negative for ova, parasites, or pathogenic organisms. Blood and rectal swab cultures yielded no growth. Serological testing for HIV, HBV (HBsAg, HBc IgM/total), HCV, tuberculosis, and other common eosinophilia-inducing infections was negative, effectively ruling out infectious triggers.

#### 2.1.2 Allergic and atopic conditions

Although the patient had a known history of asthma, the extent of gastrointestinal eosinophilic infiltration (>100 eosinophils per high-power field), duodenal involvement, and systemic symptoms, including weight loss, gastrointestinal bleeding, and severe abdominal pain, exceeded the typical presentation of allergic eosinophilia. There was no history of eczema, atopic dermatitis, or recent allergen exposure. These findings made a primary atopic process unlikely.

#### 2.1.3 Autoimmune and vasculitic diseases

Autoimmune screening was unremarkable. Antineutrophil cytoplasmic antibodies (ANCA), including c-ANCA and p-ANCA, were negative, excluding eosinophilic granulomatosis with polyangiitis (EGPA/Churg-Strauss syndrome). Antinuclear antibody (ANA) testing and extractable nuclear antigen (ENA) profile were also negative, ruling out systemic autoimmune disorders such as lupus, systemic sclerosis, or Sjögren’s syndrome. The patient denied symptoms such as rash, joint pain, ulcers, or constitutional features suggestive of an autoimmune disease.

#### 2.1.4 Malignancy and lymphoproliferative disorders

Cross-sectional imaging of the neck, chest, abdomen, and pelvis revealed no lymphadenopathy, hepatosplenomegaly, or masses suggestive of lymphoma or solid tumors. Gastrointestinal biopsies showed no evidence of malignancy, crypt abscesses, or granulomas. A bone marrow biopsy performed on August 28, 2024, demonstrated trilineage hematopoiesis with a slightly hypocellular marrow, without dysplasia, increased blasts, or neoplastic infiltration.

#### 2.1.5 Drug-induced eosinophilia

The patient had been on long-term Montelukast and Theophylline therapy for asthma without recent dosage changes. No newly introduced medications were identified, aside from Dexamol, to which the patient developed a transient rash. Although this reaction was documented as a drug allergy, it was not associated with systemic eosinophilic organ involvement, and thus, drug-induced HES was considered unlikely.

#### 2.1.6 Evaluation for clonal eosinophilia

Peripheral blood smear revealed normocytic, normochromic red blood cells with mild eosinophilia and normal platelet morphology, without circulating blasts. A repeat bone marrow biopsy confirmed marked eosinophilic infiltration with preserved maturation across all hematopoietic lineages, and no features of fibrosis or dysplasia were observed. Cytogenetic analysis, including fluorescence *in situ* hybridization (FISH) for PDGFRA, PDGFRB, and FGFR1 rearrangements, was negative. There was no evidence of BCR-ABL fusion, or other genetic abnormalities associated with chronic eosinophilic leukemia or myeloproliferative neoplasms. Although serum tryptase and IgE levels were pending, the absence of clinical or histological criteria effectively excluded systemic mastocytosis or lymphoid variant HES.

### 2.2 Management and prognosis

The patient was started on prednisone at an initial dose of 1 mg/kg/day (60 mg daily) for three weeks, with a tapering regimen initiated thereafter based on symptom improvement and declining eosinophil counts. Partial symptomatic relief was achieved within 10 days, particularly in abdominal pain and appetite. However, eosinophil levels remained elevated (AEC reduced from 1800 to ~1,300 cells/μL), and gastrointestinal symptoms relapsed during tapering. As a result, azathioprine (2 mg/kg/day) was added as a steroid-sparing immunosuppressive agent. The patient remained under close monitoring, with plans for escalation to targeted biologic therapy (e.g., anti-IL-5) if future relapses occur.

## 3 Discussion

This case highlights the considerable diagnostic complexity associated with iHES, particularly when gastrointestinal manifestations dominate the clinical picture. HES is defined by persistent eosinophilia (≥1.5 × 10^9^/L for >6 months), no identifiable secondary cause, and evidence of end-organ damage resulting from eosinophilic infiltration (1–4). The systematic exclusion of secondary and clonal causes of eosinophilia, as recommended by recent WHO guidelines (16), was crucial in establishing a diagnosis of idiopathic HES in this patient. Despite the presence of asthma, the severe gastrointestinal symptoms, histological burden of eosinophils, and lack of allergic, autoimmune, infectious, or neoplastic causes supported a primary diagnosis of iHES. The normal cytogenetics and absence of clonality further confirmed this. Although rare, HES is presented in varied clinical forms and can affect multiple organs, which complicates and often delays diagnosis.

Our patient initially presented with gastrointestinal symptoms such as epigastric pain, rectal bleeding, bowel habit alterations, and weight loss which led clinicians to consider more common diagnoses such as inflammatory bowel disease (IBD) or infectious colitis. The presence of elevated CRP and fecal calprotectin and endoscopic mucosal changes supported this initial hypothesis. However, lack of classic imaging findings, absence of granulomas or transmural inflammation on histopathology, and non-response to immunosuppressants eventually called the diagnosis into question.

Eosinophilic gastroenteritis (EGE) became a leading consideration following biopsy findings of dense eosinophilic infiltration (>100 eosinophils/HPF) in the gastric and colonic mucosa, as well as mast cell aggregates, which are sometimes seen in eosinophilic disorders (17). However, this diagnosis alone could not explain the peripheral and gastrointestinal eosinophilia, as well as the pulmonary abnormalities (lung nodules, bronchiectasis) found on CT imaging, features that are not characteristic of localized EGE (18). HES, particularly idiopathic HES, remains a diagnosis of exclusion. It requires comprehensive investigation to rule out secondary causes, including parasitic infections, drug reactions, systemic vasculitis (e.g., eosinophilic granulomatosis with polyangiitis), and clonal hematologic disorders (6, 19, 20). In this case, extensive autoimmune and infectious panels, including C-ANCA, p-ANCA, and stool examinations, were negative. The turning point was a bone marrow biopsy that confirmed marked eosinophilia with normal trilineage maturation and no evidence of myeloproliferative neoplasm or malignancy, fulfilling the diagnostic criteria for idiopathic HES (5).

This case exhibited several uncommon and diagnostically confusing features: (1) Severe gastrointestinal involvement, such as giant gastric folds and duodenitis, is rare in idiopathic HES but may occur in gastrointestinal variants of the disease (21). (2) Pulmonary changes without overt respiratory symptoms, which can indicate subclinical eosinophilic involvement of the lungs, are typical of systemic HES (22). (3) The absence of early eosinophil quantification delayed the recognition of a primary eosinophilic disorder, a common oversight in similar cases (11). Untreated HES has a historically poor prognosis, with median survival ranging from 1 to 3 years, primarily due to cardiac complications, including endomyocardial fibrosis and thromboembolic events (2). However, prompt initiation of therapy, especially systemic corticosteroids, significantly improves outcomes, with 5-year survival rates now exceeding 80% in many cohorts (8, 23). Immunomodulators such as azathioprine or biologic agents may be considered in steroid-refractory or relapsing cases (24).

## 4 Conclusion

This case illustrates the diagnostic complexity involved in distinguishing eosinophilic gastrointestinal disorders from systemic eosinophilic syndromes such as iHES. The patient initially presented with symptoms suggestive of inflammatory bowel disease and eosinophilic gastroenteritis, supported by elevated fecal calprotectin and gastrointestinal eosinophilic infiltration. However, the persistence of high-grade peripheral eosinophilia, pulmonary involvement, and absence of allergic or infectious causes prompted further investigation. Ultimately, the diagnosis of iHES was established based on WHO 2024 criteria after exclusion of reactive, autoimmune, and clonal causes. The case highlights the importance of a systematic diagnostic approach, the utility of repeated tissue and hematological evaluation, and the need for individualized long-term management. A multidisciplinary follow-up remains essential given the risk of relapse and multi-organ progression.

## Data Availability

The original contributions presented in the study are included in the article/supplementary material, further inquiries can be directed to the corresponding author.
